# The Immunomodulation Potential of Exosomes in Tumor Microenvironment

**DOI:** 10.1155/2021/3710372

**Published:** 2021-09-27

**Authors:** Meng Wang, Bo Zhang

**Affiliations:** Reproductive Medicine Center, Tongji Hospital, Tongji Medical College, Huazhong University of Science and Technology, Wuhan, China

## Abstract

Exosomes are lipid bilayer particles that originated from almost all types of cells and play an important role in intercellular communication. Tumor-derived exosomes contain large amounts of noncoding RNA, DNA, and proteins, which can be transferred into recipient cells as functional components in exosomes. These exosomal functional constituents depend on the originating cells, and it has been proved that types and numbers of exosomal components differ in cancer patients and healthy individuals. This review summarizes the role of tumor-derived exosomes in immunomodulation and discusses the application of exosomes in immunotherapy in cancers. Overall, exosomes isolated from cancer cells are turned out to promote immune evasion and interfere with immune responses in tumors through inducing apoptosis of CD8+ T cells, facilitating generation of Tregs, suppressing natural killer (NK) cell cytotoxicity, inhibiting maturation and differentiation of monocyte, and enhancing suppressive function of myeloid-derived suppressor cells (MDSCs). Mechanistically, exosomal functional components play a significant role in the immunomodulation in cancers. Moreover, based on the existing studies, exosomes could potentially serve as therapeutic delivery vehicles, noninvasive biomarkers, and immunotherapeutic vaccines for various types of cancers.

## 1. Introduction

Cancer is a global public health problem, and the incidence and mortality of cancer are dramatically increasing. It is reported that there are more than 18 million new cases and 9 million deaths each year worldwide [1–3]. There are several strategies for tumor development and survival, and tumor immune evasion, as one of the important mechanisms, enables tumors to escape from immune surveillance, inhibit antitumor immune responses, and finally, grow progressively [4]. Immune cells in tumor microenvironment play important roles in tumorigenesis [5], and exosomes released into tumor microenvironment have also been proved to be able to regulate immune responses in tumors [6].

Extracellular vesicle is membrane-bound vesicle in all body fluids for crosstalk between cells. Exosome, a subtype of extracellular vesicle, refers to lipid bilayer particles with a diameter of 50-150 mm [7]. They are released by almost all types of cells into microenvironment and have emerged as a novel method for intercellular communication through either functional component transfer or membrane receptor-mediated signaling transduction [8]. Cancer-derived exosomes are widely distributed in body fluids from tumor-xenograft animal models and cancer patients, such as plasma, ascites, pleural effusion, and other fluids [9]. It has been demonstrated that types and numbers of exosomal constituents differ in cancer patients and healthy individuals [10]. Exosomes isolated from cancer cells can interfere with immune responses and correlate with the development and progression of tumors, including tumor growth, invasion, and metastasis [11, 12]. However, the intercellular communication of cancer cells and noncancer cells via exosomes has not been fully illustrated. Therefore, this review is aimed at summarizing the functional components in exosomes, reviewing the role of tumor-derived exosomes in immunomodulation, and discussing the application of exosomes in immunotherapy in cancers.

## 2. Formation and Contents of Cancer Exosomes

**Figure 1:**
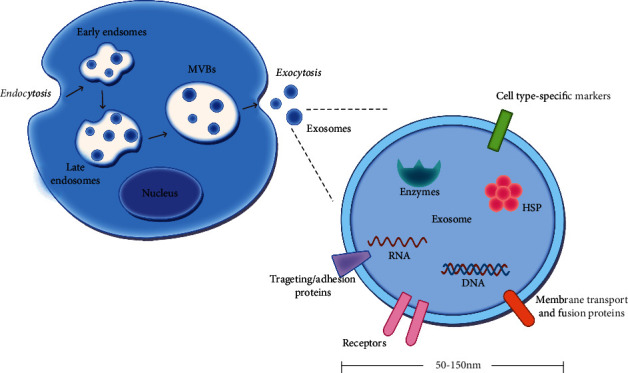
Formation and contents of exosomes. The biogenesis of exosomes is attributed to endocytosis and exocytosis of cells. The endocytosis of cell membrane can form early endosomes, which then develop into late endosomes. The process of budding of late endosomes into their lumen is limited, resulting in vesicle enrichment in internal lumen in multivesicular bodies (MVBs). Subsequently, exosomes, with a diameter of 50-150 nm, are released into extracellular space mediated by the exocytic fusion of the external membrane of MVBs and the cell membrane. The molecular compositions of exosomes are dependent on the originating cells, and structurally, exosomes are lipid bilayer vehicles with proteins and nucleic acids. MVBs: multivesicular bodies; HSP: heat shock proteins.

The biogenesis of exosomes is attributed to endocytosis and exocytosis of all cell types [13]. The endocytosis of cell membrane can form early endosomes, which are located near the cell membrane with a tubular appearance. After acidification, protein contents change, and fusion with other membrane components and early endosomes develop into late endosomes, which are located near the nucleus and exhibit a spherical shape. The multivesicular bodies (MVBs) then develop from late endosomes through reversed budding. The process of budding of late endosomes into their lumen is limited, resulting in vesicle enrichment in internal lumen [14]. Subsequently, the vesicles, also named exosomes, are released into extracellular space mediated by the exocytic fusion of the external membrane of MVBs and the cell membrane (Figure 1).

Since exosomes are originated from endocytosis and exocytosis of membrane vesicles, the molecular compositions of exosomes are dependent on the originating cells (Figure 1). Structurally, exosomes are with a lipid bilayer that encloses cytoplasm; thus, proteins and nucleic acids, main components in cytoplasm, are also the contents of exosomes (Table 1). Cancer exosomes regulate tumor biological processes, including tumor progression, metastasis, and angiogenesis.

### 2.1. RNAs in Cancer Exosomes

MicroRNA (miRNA) in cancer exosomes has been demonstrated to be involved in tumor progression. miRNAs, which inhibit targeted mRNA translation process, can be absorbed by cells through exosomes and act as tumor promoters or suppressors, finally interfering with other stromal cells in tumor microenvironment [15]. In breast cancer, tumor-derived exosomes have been proved to be specifically enriched in miRNA, which is able to stimulate a transformation of nontumor-originated breast cells into tumors [16]. Similarly, miR-105 secreted by exosomes isolated from the same type of cancer cells is reported to promote metastasis by destroying vascular endothelial barriers [17]. High levels of miR-122 in exosomes can also reprogram glucose metabolism in nontumor cells to facilitate metastasis [18]. Except for the significant role in tumor development and metastasis, miRNA may also serve as biomarkers in cancer, especially colorectal cancer [19]. A study has demonstrated that exosomal miR-320d in serum may act as a noninvasive diagnostic marker for metastatic colorectal cancer [20]. Also, the expression level of circulating exosomal miR-25-3p is also associated with the metastasis of colorectal cancer [21]. In addition, exosomal miRNAs are also significant in other types of tumors, such as esophageal squamous cell carcinoma [22] and breast cancer [15], and serve as molecular biomarkers [23].

Long noncoding RNA (lncRNA), another type of RNA content in cancer exosomes [24], also participates in biological processes of genetic transcription, mRNA translation, and protein modification [25]. It has been proved that lncRNA from cancer cell-originated exosomes is able to promote tumorigenesis by facilitating angiogenesis, suppressing immune functions, and inducing metastasis [26]. lncRNA HOTAIR can be conveyed into endothelial cells via exosomes secreted by glioma cells, and HOTAIR is known to promote angiogenesis [27]. Similarly, lncRNA H19, which facilitates hepatocarcinogenesis [28], is enriched in exosomes from liver cancer cells and can be internalized by endothelial cells, resulting in cell adhesion and angiogenesis [29]. Moreover, exosomal lncRNA SNHG16 in breast cancer is reported to suppress immune functions by inducing CD73^+^*γδ*1 Treg cells [30], revealing a close relationship between exosomal lncRNA and immunosuppression in cancers. Furthermore, many studies have also confirmed the involvement of exosomal lncRNA in tumor metastasis in different cancer types. lncRNA MALAT-1 is abundant in exosomes isolated from the serum of non-small-cell lung cancer patients, and it facilitates tumor migration and lymphatic node metastasis [31]. Meanwhile, serous exosomal lncRNA 91H, which is proved to promote tumor migration, also dramatically decreases after operation in colorectal cancer patients, indicating the significance of exosomal lncRNA in cancers.

Although circRNA is a novel type of noncoding RNA, the role of exosomal circRNA in cancer tissues has also been emphasized. The presence of circular RNA (circRNA) has been demonstrated to play a critical role in cancer growth and metastasis. Exosomal circPACRGL is upregulated in colorectal cancer, and promotes tumor cell proliferation, migration, and invasion *in vitro* [32]. Exosomal circRNAs are also found to promote cancer progression in gastric cancer [33] and tumor metastasis in hepatocellular carcinoma [34].

### 2.2. DNAs in Cancer Exosomes

Recently, it has been reported that DNA also exists in cancer-derived exosomes [35, 36], and exosomal DNA may be crucial in cell communication as RNA components do. Although the exact mechanism of DNA presence in exosomes is still under investigation, mitochondrial DNA (mtDNA) was observed in exosomes isolated from astrocytes and glioblastoma cells for the first time [37]. Interestingly, a large abundance of mtDNA can be detected on the surface of exosomes [38], and it is speculated that it may be associated with exosomal aggregation and recipient cell dysfunction [39]. Moreover, other types of DNA, including single-stranded DNA (ssDNA) and double-stranded DNA (dsDNA), are also overserved in exosomes derived from cancer cells in recent years [40, 41]. What should be noted is that exosomal dsDNA exhibits a tumor-specific manner, whose amount is much higher originated from cancer cells than normal cells [42]. It revealed that exosomal DNA may act as a noninvasion biomarker for early cancer detection. Moreover, exosomal DNA in cancers also allows for treatment monitoring. The horizontal transfer of mtDNA from exosomes can result in therapy resistance in oxidative phosphorylation-dependent breast cancer and lead to metastasis [43]. However, in another study, the so-called exosomal DNA is just a nucleic acid-histone complex resulting from autophagy [44]. The presence of exosomal DNA needs to be further investigated and explored.

### 2.3. Proteins in Cancer Exosomes

The protein components in cancer exosomes are complicated and have an endocytic origin. A recent study identified 232 unique proteins via exosomal proteome using ion trap mass spectrometry [45]. Generally, targeting/adhesion proteins, membrane transport and fusion proteins, heat shock proteins, enzymes, receptor proteins, cell type-specific markers of the originating cells are the main constitutive protein components of exosomes. In hepatocarcinoma, exosomal proteins are associated with cell migration, invasion, and angiogenesis [46]. Threspanin-8 in gastric cancer cell-derived exosomes can act as a biomarker for cancer growth and metastasis [47]. Another study also reported that exosomal CD317 and EGFR might also be used as early biomarkers for non-small-cell lung cancer [48]. Some tumor-specific markers, such as prostate-specific antigen (PSA) and survivin, have also been reported to highly express in exosomes, revealing a clinical significance of exosomal markers in diagnosis and differential diagnosis in prostate cancer [49]. In addition, the function of exosomal proteins from metastatic and nonmetastatic murine breast cancer cells is totally different. The former one is mainly involved in cell proliferation, migration, and angiogenesis, while the latter mainly participated in cell adhesion [50]. Moreover, immunosuppressive proteins are highly expressed in melanoma cell-derived exosomes, resulting in cell apoptosis, T cells proliferation suppression, and NK cells dysfunction [51]. These results emphasize that exosomal proteins in cancer play a critical role in tumor progression via immunomodulation.

## 3. Cancer Exosomes in Immunomodulation

### 3.1. Cancer Exosomes Induce Apoptosis of CD8^+^ T Cells

**Figure 2:**
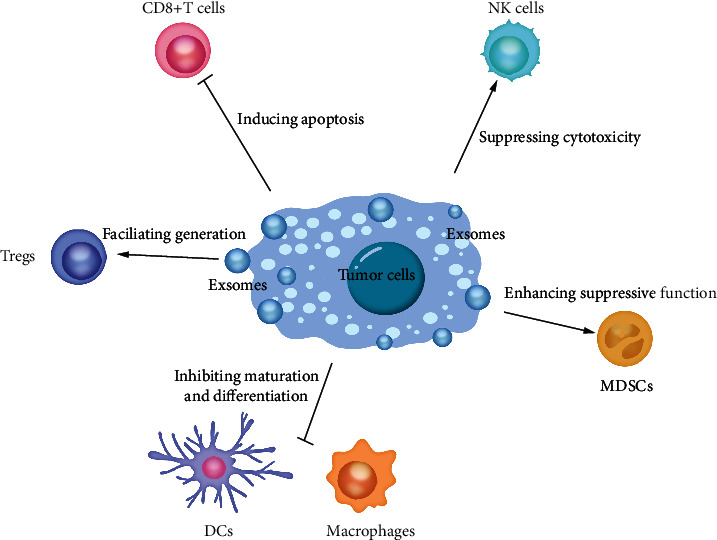
The functions of cancer exosomes in immunomodulation. Exosomes isolated from cancer cells are turned out to promote immune evasion and interfere with immune responses in tumors through inducing apoptosis of CD8+ T cells, facilitating generation of Tregs, suppressing NK cells cytotoxicity, inhibiting maturation and differentiation of monocyte, and enhancing suppressive function of MDSCs. NK cells: natural killer cells; DCs: dendritic cells; MDSCs: myeloid-derived suppressor cells.

Cytotoxic CD8^+^ T cells and CD4^+^ Th1 cells are the main antitumor immune response effector cells [52, 53]. Cancer-derived exosomes regulate functions of T cells mainly through impairing proliferation and facilitating apoptosis of CD8^+^ T cells [54], while immune cell-derived exosomes intend to promote T cell proliferation [55] (Figure 2). Fas ligand (FasL) has been reported to be highly expressed in various types of tumor cell-derived exosomes, including ovarian cancer [56], melanoma [54], prostate cancer [57], and oral squamous cell carcinoma [58]. The activation of Fas/FasL signaling pathway can promote apoptosis and downregulate immune response [59]. Several studies have also demonstrated that FasL-expressed cancer exosomes can induce CD8^+^ T cell apoptosis and be correlated with poor prognosis [54, 60]. The interaction between programmed death-ligand 1 (PD-L1) in cancer cells and programmed cell death protein 1 (PD-1) receptor in activated T cells is another mechanism of immune evasion in cancer [61]. On the one hand, exosomal PD-L1 can inhibit T cell activation and proliferation [62]; on the other hand, it can be transferred into cancer cells [63], finally resulting in immunosuppression amplification. In non-small-cell lung cancer, the exosomal PD-L1 level in serum is much higher in patients suffering from cancers with a higher TMN stage, indicating that the level of exosomal PD-L1 might be a potential cancer progression monitoring [64]. Similarly, exosomal PD-L1 expression in serum is also regarded as a reliable marker for treatment response prediction in melanoma [61]. Apart from FasL and PD-L1, it is also reported that exosomes can induce immunosuppression through many mechanisms. For instance, galectin-9 is detected in exosomes isolated from nasopharyngeal carcinoma patients. The binding of exosomal galectin-9 and mucin-domain containing-3 (Tim-3) in T cells promotes apoptosis of CD4^+^ T cells, and this process can be reserved by neutralizing these two components [65].

### 3.2. Cancer Exosomes Facilitate Generation of Tregs

Regulation T cells (Tregs) help tumor cells escape from attacks of immune system by releasing immunosuppressive cytokines, such as IL-10 and TGF-*β*1 [66], and the expression of tumor-infiltrating Tregs has a close relationship with the prognosis of cancer. It has been demonstrated that exosomes isolated from tumor cells facilitate generation and expansion of Tregs [67] (Figure 2). CD4^+^ CD25^−^ T cells can be converted into CD4^+^ CD25^high^ FoxP3^+^ Tregs after coculture with tumor-derived exosomes. In terms of mechanism, exosomal miR-214 inhibits the expression of phosphatase and tensin homolog (PTEN) in T cells and induce Tregs to secret IL-10, finally promoting tumor growth [68]. Similar phenomena can also be investigated in another study. Exosomes isolated from mutant KRAS lung cancer cells are found to facilitate the switch of naïve CD4^+^ T cells into Tregs, which is also observed after the transfection of mutant KRAS cDNA. It indicates that exosomal mutant KRAS DNA leads to this conversion, and it is further confirmed by the enrichment of FoxP3^+^ Tregs in tumor tissues from mutant KRAS patients, compared to the WT KRAS controls [69]. Moreover, lncRNA SNHG16 transmitted by exosomes is also proved to induce CD73^+^*γδ*1 Treg in breast cancer by sponging miR-16-4p, which enables the downregulation of SMAD5, the subsequent enhancement of TGF-b1/SMAD5 pathway, and finally, the promotion of CD73 expression [30]. Furthermore, Th17 cells can either induce angiogenesis and immunosuppression to facilitate tumor progression or recruit immune cells to promote antitumor immune response [70]. An upregulated Treg/Th17 ratio can be observed in ovarian cancer patients, and the imbalance of Treg/Th17 is induced by the transfer of miR-29a-3p and miR-21-5p mediated by cancer-derived exosomes. These exosomal miRNAs can directly induce signal transducer and activator of transcription 3 (STAT3) inhibition, promoting cancer progression and metastasis [71].

### 3.3. Cancer Exosomes Suppress Cytotoxicity of NK Cells

Natural killer (NK) cells are innate lymphocytes involved in antitumor immune response and immune surveillance [72]. Natural killer group 2 member D (NKG2D) is an activating receptor for NK cells, loss of which is crucial in immune evasion [73]. It is found that the activity of NK cells is frequently decreased in cancer patients compared with healthy controls [74], so the expression of NKG2D in NK cells treated with exosomes isolated from cancer cells [75] (Figure 2). Studies have demonstrated that cancer-derived exosomes express NKG2D ligands to depress NKG2D expression and inhibit NK cell cytotoxicity [76]. The presence of TGF-*β*1, as a cytokine that can suppress NK cells cytotoxicity, in tumor-derived exosomes may contribute to the suppression of NK cells activity in cancers [75], which can be verified by the rescue of exosome-mediated NKG2D reduction in NK cells upon TGF-*β*1 neutralization [74]. Some other studies have also suggested that exosomes from cancers can promote tumor growth by impairing NK functions [77]. Noncoding RNAs in exosomes, such as circUHRF1, are also proved to be associated with NK cell exhaustion in cancers [78].

### 3.4. Cancer Exosomes Inhibit Maturation and Differentiation of Monocyte

Monocytes are innate myeloid cells that can differentiate into macrophages and dendritic cells (DCs). Cancer exosomes have been proved to induce immunosuppression by impairing maturation and differentiation of monocytes [79] (Figure 2). DCs, as specialized antigen-presenting cells, have significant functions in innate and adaptive immune responses. However, DC functions are suppressed in tumor environment, and thereby immune-suppressive DCs are recruited and infiltrated in tumor tissues [80]. Cancer exosomes are first reported to inhibit monocyte differentiation by Valenti et al. [81]. Exosomes from lung and breast cancers block the process of myeloid precursor cells into DCs [82]. It has been reported that exosomes isolated from colorectal cancer and melanoma cells inhibit the differentiation of monocytes to DCs [81]. The possible mechanism of the suppression in immune system may attribute to the protein components in exosomes, such as TGF-*β*, IL-6, and PGE2 [83]. It has been demonstrated that tumor-derived exosomes lead to inhibition of the differentiation of bone marrow myeloid precursors into DCs via secreting IL-6 and activating Stata3 signaling [84]. The intake of exosomal TGF-*β*1 released by tumor cells by immature DCs can also block DC maturation [85]. Meanwhile, the differentiation of monocytes to macrophages in colorectal cancer can also be regulated by exosomes [86]. Exosomes from glioblastoma-derived stem cells can foster the differentiation of monocytes to M2 macrophages, resulting in suppression in immune response [87]. Detailed mechanisms may focus on noncoding RNA in exosomes. For instance, the levels of miR-222-3p released by exosomes are higher in ovarian cancer patients, and it can be transferred into macrophages to induce tumor-promoting M2 population, resulting in tumor growth promotion [88]. Exosomal miR-200b is also upregulated in serums of ovarian cancer patients and promotes proliferation and invasion of cancer cells via inducing M2 macrophage polarization [89]. Immunosuppressive monocytes are gained by the fusion of tumor-derived exosomes and monocytes, exhibiting as high CD14 expression without HLA-DA expression [90], and CD14^+^HLA-DR^lo/neg^ monocytes, as tumor-induced immunosuppressive mediator, have been proved to increase in serum of many cancers, such as pancreatic cancer [91]. Moreover, horizon transfer of tumor antigens into antigen-presenting cells, such as monocytes, macrophages, and DCs mediated by tumor-derived exosomes may activate tumor immune response, interfering with processes of intercellular communication [92]. In summary, tumor-derived exosomes mediate immunosuppression by interfering with the differentiation of monocytes into DCs and macrophages.

### 3.5. Cancer Exosomes Enhance Suppressive Function of MDSCs

Myeloid-derived suppressor cells (MDSCs), equipped with strong immune suppressive activity, are immature myeloid cells with multiple phenotypes in tumor microenvironment [93]. The accumulation of MDSCs negatively impacts the process of antigen processing and presentation and produces abundant immunosuppressive factors to interfere with immune responses [94]. Several studies have indicated that the suppressive function of MDSCs on T cells can be potentiated by cancer exosomes (Figure 2). In renal cancer, exosomal HSP70 facilitates proliferation and enhances activation of MDSCs via activating TLR2 signaling [95]. Meanwhile, miR-9 and miR-181a from breast cancer-derived exosomes facilitate tumor growth and immune evasion via enhancing MDSC function through activating JAK/STAT signaling pathway by separately inhibiting SOCS3 and PIAS3 [96]. TGF-*β*1 and PGE2 from exosomes isolated from breast cancers help with the accumulation of MDSCs and the enhancement of tumor growth [97]. Similarly, under hypoxia conditions, miR-10a and miR-21 in glioma-derived exosomes mediate MDSC expansion and activation via RORA and PTEN, respectively [98]. Exosomal miR-21 also plays a significant role in the modulation of MDSC function in oral squamous cell carcinoma microenvironment. Exosomes derived from hypoxic oral squamous cell carcinoma enhance immunosuppressive function of MDSCs to interfere with moderation functions of *γδ* T cell via miR-21/PTEN/PD-L1 signaling pathway [99].

## 4. Cancer Exosomes in Immunotherapy

Immunotherapy has become a popular therapeutic option for cancer patients. Exosomes, membranous vesicles secreted by almost all types of cells, can be absorbed and internalized by recipient cells via membrane fusion, receptor transportation, and many other pathways [100]. Given the excellent modifiability, biocompatibility, and cyclic half-life, exosomes are regarded as potential therapeutic delivery vehicles for biological components such as antibodies and chemotherapeutic drugs [101, 102]. Abundant tumor peptide antigens, such as MHC I and MHC II, are capsulized in exosomes and can be used to stimulate antitumor responses as cell-free vaccines. In animal models, administration of exosomes-loaded DCs can improve the therapeutic effect of cytotoxic drugs and prolong the survival time [103]. To improve the targeting efficiency of naturally occurring exosomes, exosome reprogramming is becoming popular. A novel exosome platform named synthetic multivalent antibodies retargeted exosome (SMART-Exo) is designed and developed to enable exosome genetic modification [104, 105]. Exosomes are reprogrammed to express CD3-specific antibodies for T cells and EGFR antibodies for EGFR-expressing breast cancer cells [104]. In another study, exosomes are also engineered to display both anti-CD3 and anti-HER2 antibodies to target cytotoxic T cells and HER2-expressing breast cancer cells [105]. In addition to breast cancer, CD80 and CD86 are also packed in engineered exosomes to facilitate immune responses and secret immune-associated cytokines in leukemia [106]. These studies demonstrate that exosome reprogramming might be potential targeted immunotherapy for cancers. Another exosome-based drug delivery system named exosome-based superparamagnetic nanoparticle cluster (SMNC-EXO) is also developed to help exosomes with targeted drug delivery in the presence of external magnetic fields [107]. In a word, engineered exosome-based drug delivery systems might be a promising candidate for antitumor immunotherapy in the future (Table 2).

It has been shown that activated immune cells can secret exosomes containing miRNAs that can be used as biomarkers for immunotherapy. A study investigates exosomal miRNA profiles in non-small-cell lung cancer patients receiving PD-1/PD-L1 immunotherapy and healthy controls. More than 150 unique exosomal miRNAs are identified in cancer patients, and hsa-miR-320d, hsa-miR-320c, and hsa-miR-320b may be potential biomarkers to predict treatment efficacy of PD-1/PD-L1 immunotherapy in lung cancers [108]. The levels of plasma exosomal caveolin-1 are found to be downregulated in ovarian cancer, and they are positively associated with prognosis and overall survival as a biomarker [109]. Similarly, plasma-derived exosomal miR-4732-5p is also highly expressed in epithelial ovarian cancer patients and could be used to monitor cancer progression [110]. The above studies suggest the significance of exosomes as biomarkers in immunotherapy and prognosis monitor.

However, most of the current studies that investigated the predictor value of exosomal components in cancer progression and immunotherapy are laboratory studies; the clinical values should be confirmed in clinical trials. The first exosome phase I trial reported the administration of autologous exosomes derived from DCs in 15 cancer patients, and the results confirm the safety of exosome administration, despite the fact that no specific T cell responses are observed in circulating [111]. Meanwhile, some other clinical trials also prove that plasma DC-derived exosomes in cancer patients are also proved to be effective. It is worthy noted that although exosomes are expected to be potential immunotherapeutic vaccines in cancer treatment, it still will be a long time for exosome-based immunotherapy in clinical practices [112].

## 5. Challenge in Clinical Application of Exosomes for Cancer Treatment

It seems that exosomes isolated from tumors have an excellent prospect and are emerged as a potential tool for cancer therapy, yet the future of exosomes in clinical application still present many challenges. Currently, there is no gold standard for exosome isolation and purification, but new methodologies seemingly emerge in an endless stream. It has been reported that the purity and concentration of exosomes isolated vary with isolation methods [113]. A comparison of miRNA profiles of exosomes isolated by different methods suggested that the discrepancies of the content and amount of miRNA result from methodological differences [114]. Similar results are also reported in other studies, in which the circulating exosomal miRNA varies due to the isolated methods used [115]. Moreover, the proteins in exosomes may also be differential in results of the difference of isolation techniques [116]. Ultracentrifugation is the most widely used approach for exosome isolation worldwide [117]; however, several shortcomings, including the existence of nonexosomal impurity, potential damage, low yield of exosomes, and RNA components, are still investigated [118]. In addition to isolation methods, concentration and components of exosomes can also be influenced by microenvironments. It has been found that hypoxic conditions can increase the production of exosomes [119]. Meanwhile, exosomal miRNAs are also reported to be influenced by the oxygen concentration in microenvironment in terms of expression level [120]. The inconsistency of productions and the drawbacks in different isolation methods limit the clinical utilization of exosomes in diagnostic, prognostic, and therapeutic applications. Furthermore, although some components in exosomes derived from cancer cells, such miRNAs and proteins, are thought to be potential biomarkers for diagnosis and prognosis of cancers [121–123], these biomarkers are lack of specificity on screening, which may narrow their application. Focusing on the limitations of characterizations of exosomes as well as isolation methods is conductive to the development of exosomes in human diseases.

## 6. Conclusion

Tumor-derived exosomes contain large amounts of noncoding RNA, DNA, and proteins, which can be transferred into recipient cells as functional components in exosomes. Exosomes isolated from cancer cells are turned out to promote immune evasion and interfere with immune responses in tumors through inducing apoptosis of CD8+ T cells, facilitating generation of Tregs, suppressing NK cells cytotoxicity, inhibiting maturation and differentiation of monocyte, and enhancing suppressive function of MDSCs. Mechanistically, exosomal functional components play a significant role in the immunomodulation in cancers. Moreover, based on the existing studies, exosomes could potentially serve as therapeutic delivery vehicles, noninvasive biomarkers, and immunotherapeutic vaccines for various types of cancers.

## Figures and Tables

**Table 1:** Contents of cancer exosomes.

Content	Molecular types	Function
RNA	MicroRNA (miRNA)	Inducing metastasis, invasion, and transformation and formation of tumors in noncancerous cells; serving as biomarkers in cancers
	Long noncoding RNA (IncRNA)	Promoting tumorigenesis by facilitating angiogenesis, suppressing immune functions, and inducing metastasis
	Circular RNA (circRNA)	Promoting carcinoma growth and metastasis
DNA	DNA in exosomes (exoDNA)	Being associated with abnormal DNA replication in cancer cells, and reverse transcription of cellular RNA; mediating immune response activation
Protein	Targeting/adhesion proteins	Regulating tumor proliferation, growth, metastasis, migration, angiogenesis, adhesion, immune suppression, and many other biological processes of tumor; having potential immunotherapeutic effects
	Membrane transport and fusion proteins	
	Heat shock proteins	
	Enzymes	
	Receptor proteins	
	Cell type-specific markers of the originating cells	

**Table 2:** Engineered exosomes for cancer immunotherapy.

Exosome types	Exosome source	Functional cargo	Immune response	Reference
SMART-Exo	Expi293F	Anti-CD3, anti-HER2, anti-EGFR	Activating and redirecting T cells toward HER2- or EGFR-expressing breast cancer cells	[105]
B7-1 and B7-2 Exo	Leukemia cells	B7 costimulatory proteins and leukemia-associated antigens	Facilitating T cell-mediated antitumor responses	[106]
Cell-free vaccine	DCs differentiated from autologous monocytes	MHC and antigenic peptide	Initiating T cell responses	[124]
CD40L-Exo	3LL Lewis lung cells	CD40L, TAA	Activating DCs-mediated antitumor immunity in 3LL tumors	[125]
TEX-N1ND	HCC, breast and pancreatic cancer cells	N1ND and TAA	Activating DCs-mediated antitumor immune response	[126]
IFN*γ*-Exo vaccine	RM-1 cancer cells	IFN-*γ* and TAA	Activating M1-mediated antitumor immune response in RM-1 tumors	[127]
Decoy for TNF-*α*	HEK293	Extracellular domain of TNFR1	Antagonizing TNF-*α in vitro*	[128]

SMART-Exo: synthetic multivalent antibodies retargeted exosome; HER2: human epidermal growth factor receptor 2; EGFR: epidermal growth factor receptor; DCs: dendritic cells; MHC: major histocompatibility complex; CD40L: CD40 ligand; TAA: tumor-associated antigens; TEX: tumor-derived exosome; N1ND: N-terminus domain of HMGN1; HCC: hepatocellular carcinoma; IFN-*γ*: interferon gamma; M1: macrophage 1; TNFR1: tumor necrosis factor receptor 1; TNF-*α*: tumor necrosis factor alpha.
